# Biventricular dimensions and function in pediatric sickle-cell disease and thalassemia major patients without cardiac iron

**DOI:** 10.1186/1532-429X-15-S1-P111

**Published:** 2013-01-30

**Authors:** Antonella Meloni, Jon Detterich, Vasili Berdoukas, Alessia Pepe, Massimo Lombardi, Thomas D Coates, John C Wood

**Affiliations:** 1 CMR Unit, Fondazione G.Monasterio CNR-Regione Toscana and Institute of Clinical Physiology, Pisa, Italy; 2 Department of Pediatrics, Division of Cardiology, Children's Hospital Los Angeles, Los Angeles, CA, USA; 3 Division of Hematology, Children's Hospital Los Angeles, Los Angeles, CA, USA; 4 Department of Radiology, Children's Hospital Los Angeles, Los Angeles, CA, USA

## Background

Chronically anemic patients develop compensatory ventricular dilation, even when maintained on chronic transfusion regimens. Our primary goal was to compare right and left ventricular dimensions and function assessed by Cardiovascular Magnetic Resonance (CMR) in pediatric, chronically-transfused sickle-cell disease (SCD) and thalassemia major (TM) patients who lacked cardiac iron. Moreover we explored systematic sex differences in ventricular dimensions in both populations.

## Methods

We reviewed all CMRs identifying 261 studies suitable for analysis from 64 SCD patients (34 females and 30 males) and 49 TM patients (29 males and 20 females). All demographic and CMR parameters were inversely weighted by the number of exams. Analysis of covariance (ANCOVA) models were used to evaluate the impact of potential covariates (variables unbalanced between groups and associated with the outcome) on group differences in CMR parameters.

## Results

In both populations, males had larger left ventricular (LV) and right ventricular (RV) dimensions than females, with a more marked effect observed in SCD patients. The percentage difference for the RV was larger than that one seen in normal subjects (from 8 to 14%). Table 1 shows the comparison of LV parameters between SCD and TM with the differentiation by sex. All LV volumes as well as the LV mass were significantly higher in SCD than in TM patients, also adjusting for the covariates. Table 2 shows the comparison of RV parameters between SCD and TM, by sex. Overall findings are similar to those from the LV. All RV volumes remained significantly higher in SCD also after ANCOVA adjustments, except RV ESVI.

**Table 1:** LV parameters for and SCD and TM pediatric patient without cardiac iron overload, with differentiation for gender. All variables are expressed as mean ± SD with 95% confidence intervals in square brackets.

	SCD	TM	P	P adjusted for systolic BP	P adjusted for cardiac R2*	P adjusted for both covariates
***Males***						

**LV EDVI (ml/m^2^)**	104.7 ± 15.2 [99.5 - 109.9]	88.7 ± 11.0 [84.5 - 92.8]	<0.0001	<0.0001	<0.0001	<0.0001

**LV ESVI (ml/m^2^)**	38.1 ± 7.4 [35.6 - 40.7]	31.6 ± 5.5 [29.5 - 33.6]	<0.0001	<0.0001	0.0003	<0.0001

**LV SVI (ml/m^2^)**	66.6 ± 9.5 [63.3 - 69.8]	57.1 ± 6.5 [54.6 - 59.5]	<0.0001	<0.0001	<0.0001	<0.0001

**LV EF (%)**	63.8 ± 3.5 [62.6 - 65.0]	64.7 ± 2.8 [63.6 - 65.7]	0.286			

**LV MI (g/m^2^)**	83.5 ± 11.4 [79.1 - 87.9] (27 pts; 45 MRIs)	66.9 ± 7.0 [64.0 - 69.8] (24 pts; 59 MRIs)	<0.0001	<0.0001	<0.0001	<0.0001

**LV CI (l/min/m2)**	4.8 ± 0.6 [4.6 - 5.1] (32 pts; 55 MRIs)	4.5 ± 0.5 [4.4 - 4.7] (29 pts; 74 MRIs)	0.017			

***Females***						

**LV EDVI (ml/m^2^)**	93.5 ± 9.6 [90.0 - 97.0]	81.4 ± 6.7 [78.4 - 84.4]	<0.0001	<0.0001	<0.0001	<0.0001

**LV ESVI (ml/m^2^)**	36.0 ± 5.3 [34.0 - 37.9]	29.4 ± 3.8 [27.6 - 31.1]	<0.0001	<0.0001	<0.0001	<0.0001

**LV SVI (ml/m^2^)**	57.5 ± 6.3 [55.2 - 59.8]	52.0 ± 4.2 [50.1 - 53.4]	0.0006	0.009		

**LV EF (%)**	61.6 ± 3.5 [60.4 - 62.9]	64.1 ± 2.9 [62.8 - 65.4]	0.008			

**LV MI (g/m^2^)**	72.9 ± 8.9 [69.6 - 79.3] (28 pts; 60 MRIs)	62.8 ± 7.5 [60.4 - 66.1] (18 pts; 40 MRIs)	0.0001	0.0002		

**LV CI (l/min/m^2^)**	4.6 ± 0.6 [4.4 - 4.8] (72 MRIs)	4.2 ± 0.5 [3.9 - 4.4] (52 MRIs)	0.006			

**Table 2:** RV parameters for and SCD and TM pediatric patient without cardiac iron overload, with differentiation for gender. All variables are expressed as mean ± SD with 95% confidence intervals in square brackets.

	SCD	TM	P	P adjusted for systolic BP	P adjusted for cardiac R2*	P adjusted for both covariates
***Males***						

**RV EDVI (ml/m^2^)**	103.3 ± 18.8 [96.8 - 109.8]	87.3 ± 11.5 [82.9 - 91.6]	<0.0001	<0.0001	0.0004	0.0002

**RV ESVI (ml/m^2^)**	37.9 ± 9.6 [34.7 - 41.2]	32.4 ± 5.7 [30.2 - 34.5]	0.0005	0.006	0.050	0.037

**RV SVI (ml/m^2^)**	65.4 ± 10.8 [61.6 - 69.1]	54.9 ± 6.9 [52.3 - 57.5]	<0.0001	0.016	<0.0001	<0.0001

**RV EF (%)**	63.8 ± 4.7 [62.2 - 65.4]	63.2 ± 3.4 [61.9 - 64.5]	0.620			

***Females***						

**RV EDVI (ml/m^2^)**	84.7 ± 10.3 [80.9 - 88.5]	76.5 ± 9.1 [72.4 - 80.6]	0.005	0.026	0.009	0.051

**RV ESVI (ml/m^2^)**	31.1 ± 5.8 [28.9 - 33.2]	27.7 ± 5.3 [25.3 - 30.1]	0.037	0.108		

**RV SVI (ml/m^2^)**	53.6 ± 6.8 [51.1 - 56.1]	48.8 ± 5.1 [46.6 - 51.1]	0.006	0.047		

**RV EF (%)**	63.6 ± 4.6 [61.9 - 65.2]	64.4 ± 3.7 [62.7 - 66.1]	0.477			

## Conclusions

Compared to TM patients, SCD patients showed significantly greater biventricular dilation and LV hypertrophy. This difference could not be explained by different hemoglobin levels, cardiac iron overload and systolic blood pressure. Our results represent important baseline findings that place changes introduced by iron overload as well as systemic and pulmonary vasculopathy in proper context.

## Funding

This work was supported by the NHLBI (1 RO1 HL075592-01A1), General Clinical Research Center at the Children's Hospital Los Angeles (RR000043-43), Center for Disease Control (Thalassemia Center Grant U27/CCU922106), Novartis Pharma, and the Department of Pediatrics.

